# Laparoscopic cholecystectomy performed by general surgery residents. Is it safe? How much does it cost?

**DOI:** 10.1590/0100-6991e-20202907

**Published:** 2021-05-05

**Authors:** JORGE HENRIQUE BENTO DE SOUSA, FRANCISCO TUSTUMI, MILTON STEINMAN, OSCAR FERNANDO PAVÃO DOS SANTOS

**Affiliations:** 1 - Hospital Israelita Albert Einstein, Serviço de Cirurgia Geral - São Paulo - SP - Brasil

**Keywords:** Cost Analysis, Medical Education, Medical Residence, Results Assessment, General Surgery, Laparoscopic Cholecystectomy, Análise de Custo, Educação Médica, Residência Médica, Avaliação de Resultados, Cirurgia Geral, Colecistectomia Videolaparoscópica

## Abstract

**Objective:**

to evaluate the effectiveness and safety of laparoscopic cholecystectomies performed by residents of the first and second-year of a general surgery residency program. We studied the primary total cost of treatment and complication rates as primary outcomes, comparing the groups operated by senior and resident surgeons*.*

**Methods:**

this was a retrospective cohort study of patients who underwent laparoscopic cholecystectomy performed in a training hospital of large surgical volume in Brazil, in the period between June 1, 2018 and May 31, 2019. The study population comprised patients who underwent elective cholecystectomy due to uncomplicated chronic calculous cholecystitis or to the presence of gallbladder polyps with surgical indication. We divided the cases into three groups, based on the graduation of the main surgeon at the time of the procedure: first-year residents (R1), second-year residents (R2), and trained general surgeons (GS)*.*

**Results:**

during the study period, 1,052 laparoscopic cholecystectomies were performed, of which 1,035 procedures met the inclusion criteria, with 78 (7.5%) patients operated on with the participation of first-year residents (R1), 500 (48.3%) patients with the participation of second-year residents (R2), and 457 (44.2%) with the participation of senior surgeons only. There was no difference in conversion rates, complications, and reporting of adverse events between groups. We observed a significant difference regarding hospitalization costs (p = 0.003), with a higher mean for the patients operated with the participation of R1, of US$ 2,671.13, versus US$ 2,414.60 and US$ 2,396.24 for the procedures performed by senior surgeons and R2, respectively*.*

**Conclusions:**

laparoscopic cholecystectomy with the participation of residents is safe, even in their first years of training. There is an additional cost of about 10% in the treatment of patient operated with the participation of first-year residents. There was no significant difference in the cost of the group operated by second-year residents.

## RESUMO

**Objetivo:**

avaliar a efetividade e segurança da realização de colecistectomias laparoscópicas por residentes do primeiro e segundo ano do programa de cirurgia geral. Foram estudados como desfechos primários o custo médio total de tratamento e os índices de complicações, comparando os grupos operados por cirurgiões seniores e residentes.

**Métodos:**

trata-se de estudo de coorte retrospectivo de pacientes submetidos a colecistectomias laparoscópicas realizadas em hospital escola de grande volume cirúrgico, no Brasil, no período entre 01 de junho de 2018 e 31 de maio de 2019. A população do estudo compreendeu pacientes que realizaram colecistectomias eletivas por colecistite calculosa crônica não complicada ou por presença de pólipos de vesícula biliar com indicação cirúrgica. Os casos foram divididos em 3 grupos, baseados na graduação do cirurgião principal no momento do procedimento: residentes do primeiro ano (R1), residentes do segundo ano (R2) e cirurgiões formados (CG).

**Resultados:**

no período do estudo, foram realizadas 1.052 colecistectomias videolaparoscópicas, sendo que, após aplicados os critérios de exclusão, foram incluídos no estudo 1.035 procedimentos, com 78 (7,5%) pacientes operados com a participação de residentes do primeiro ano (R1), 500 (48,3%) pacientes com a participação de residentes do segundo ano (R2) e 457 (44,2%) apenas com a participação somente de cirurgiões seniores. Não houve diferença nas taxas de conversão, de complicações e de notificações de eventos adversos entre os grupos. Foi evidenciada diferença com relação aos custos de internação (p= 0,003), sendo observado maior custo médio de internação para os pacientes operados com participação dos R1, com custo médio de US$ 2.671,13, versus US$ 2.414,60 e US$ 2.396,24 das operações realizadas pelos cirurgiões seniores e R2, respectivamente.

**Conclusões:**

é segura a realização de colecistectomia videolaparoscópica com a participação de residentes, mesmo em seus primeiros anos de formação. Existe custo adicional de cerca de 10% no tratamento de pacientes operados com a participação de residentes do primeiro ano. Não foi observada diferença significativa no custo do grupo operado por residentes do segundo ano.

## INTRODUCTION

More than a century ago, William Stewart Halsted established one of the first surgery training programs, which was based on the concept of levels of responsibility of the so-called resident, based on his years of experience^1^ ^,^ ^2^. Since then, the surgical community has debated the delicate balance between medical education and patient care^3^.

The participation of residents in the operating room is a fundamental step in the training of the surgeon. Even with advances in the field of simulation, there is no substitute for practical teaching in the field^4^. Appendectomies, herniorrhaphies, and cholecystectomies are relatively simple surgical procedures that traditionally offer ample opportunity for residents to acquire basic training in their operative skills at a relatively early stage of their careers^5^. 

The replacement of open cholecystectomy by laparoscopic approach as a standard treatment for calculous gallbladder disease occurred in the early 1990s^6^. Laparoscopy is a challenging access route in terms of guidance, as supervision is often only vocal and requires major changes in operative settings for the tutor to intervene^3^. 

On the other hand, the laparoscopic approach has become the standard for many types of operations and is routinely performed, even in smaller hospitals^3^. Thus, the development of laparoscopic skills is mandatory in a general surgery residency program. In this context, laparoscopic cholecystectomy is among the first and most frequent laparoscopic experiences of the surgery resident^8^. 

In the literature, there are conflicting data regarding the increase in morbidity and mortality of patients in whose procedure there has been the participation of a resident^7^ ^-^ ^13^. In addition to issues related to patient safety, there is a common perception that the resident’s involvement may have an economic impact^9^ ^,^ ^14^ ^,^ ^15^.

## METHODS

This is a retrospective cohort study involving patients undergoing laparoscopic cholecystectomy performed in a training hospital of large surgical volume, the Vila Santa Catarina County Hospital, in the period between June 01, 2018 and May 31, 2019.

The study was approved by the Ethics in Research Committee of the Albert Einstein Israelite Hospital and the Ethics Committee of the Municipal Health Secretariat of the city of São Paulo, opinion number 3,573,549.

The study population consists of convenient, consecutive sampling of patients who underwent elective cholecystectomy due to uncomplicated chronic calculous cholecystitis or to the presence of gallbladder polyps with surgical indication. We excluded patients with choledocholithiasis, Mirizzi syndrome, or who underwent biliary exploration during the operation.

We divided the cases into three groups, based on the graduation of the main surgeon at the time of the procedure: first-year residents (R1), second-year residents (R2), and trained general surgeons (GS). In the period proposed for the study, the operations were performed by six R1, six R2, and 15 surgeons specialized in digestive tract surgery, with at least five years of experience in laparoscopy.

Cholecystectomies always started laparoscopically and were performed in a standardized manner, with intraoperative cholangiography only in selected cases (dilation of the bile ducts, anatomical doubt).

According to the institution’s protocol, all cases at increased risk for the presence of choledocholithiasis (increase in serum levels of bilirubin or canalicular enzymes, dilation of bile ducts on imaging exam, or history of pancreatitis or jaundice) underwent preoperative Magnetic Resonance Cholangiography (MRC). 

Residents were always assisted by trained surgeons and, in situations of non-progression of the operative act, whether due to technical or intrinsic difficulties, roles were reversed in the operative field, and these events were accounted for. In the group in which the operations were performed by the senior surgeons, he/she was aided by another experienced surgeon. Patients were followed for at least 30 days.

We collected demographic data on sex, age, body mass index (BMI), presence of comorbidities, anesthetic classification of the American Society of Anesthesiologists (ASA) synthesized as the Severity of Illness (SOI), presence of previous laparotomy in the upper abdomen, and preoperative resonance.

The assessed outcomes were total treatment cost per patient (individual hospital bill, collected at the hospital cost center), complications, whether intraoperative or up to 30 days (stratified according to the Clavien-Dindo classification)^16^, surgical time (defined as time between first incision and skin closure), conversion to open surgery, need for transfusion of blood products, need of intensive care (ICU), reoperations, length of hospital stay, and notifications of adverse events.

Because we were interested in operative performance and not in factors related to patients, we recorded only complications related to the technical or judgmental aspects of the operation itself. For example, we did not consider pneumonia cases in this study.

Categorical variables are represented as rates, and continuous ones, as mean ± standard deviation. We submitted all variables and outcome measures to the Shapiro-Wilk normality test to determine the type of distribution. We compared the differences of results between groups with participation of the first-year resident, second-year resident, and without the participation of residents using analysis of variance (ANOVA) test for quantitative variables with normal distribution and the Kruskal-Wallis test when the variable was not normally distributed. For qualitative variables, we used the Chi-square test or the Fisher’s exact test, when indicated. We analyzed the data with the IBM SPSS Version 26 (Armonk, NY), with p <0.05 defined as statistically significant.

## RESULTS

During the study period, 1,052 laparoscopic cholecystectomies were performed, and, after applying the exclusion criteria, were included 1,035 procedures in the study.

The demographic data of the operated patients are shown in Table 1; 850 patients were female (82.1%) and 185, male (17.9%). The mean age was 52 years and the mean BMI, 31.27 kg/m^2^. Most patients were white and brown, 481 (46.5%) and 429 (41.4%), respectively. A thousand and thirteen patients (97.30%) were classified by the Severity of Illness (SOI) as “1”, 12 (1.20%) had undergone previous laparotomies, and 140 (13.50%) required preoperative MRC.

**Table 1:** Demographic data of operated patients.

Demographic data	Values
Age ± Standard Deviation	52.0 ± 14.8
BMI ± Standard Deviation	31.279 ± 6.763
Sex n(%)	
Female	850(82.10)
Male	185(17.90)
Raça n(%)	
White	481(46.50)
Brown	429(41.40)
Black	113(10.90)
Asian	10(1)
Native-American	2(0.20)
SOI (Severity of Illness) (%)	
1	1013(97.90)
2	22(2.10)
Previous laparotomy	12(1.20)
Preop MRC	140(13.50)

Table 2 shows the outcomes. The mean hospitalization time was 1.3 days, the mean operative time was 52 minutes, and the mean total cost was US$ 1,636.10. There were eight (0.77%) conversions to open procedure, 33 (3.19%) Clavien complications classified as I and II, three (0.29%) Clavien complications III, IV and V, 11 (1.06%) intraoperative complications, 25 (2.42%) postoperative complications, two (0.19%) blood transfusions, 19 (1.84%) patients in need of ICU, and 40 (3.86%) notifications of adverse events. There were no deaths during the study period.

**Table 2:** Outcomes.

Outcomes	Mean(SD) or n(%)
Hospitalization days mean± Standard Deviation	1.23 ± 1.16
Surgery time (minutes) mean± Standard Deviation	52.0 ± 24.3
Total cost mean± Standard Deviation	6.299.10 ± 2.477.20
Conversions n(%)	8(0.77)
Complications Clavien I and II n(%)	33(3.19)
Complications Clavien III, IV and V n(%)	3(0.29)
Intraop. complications n(%)	11(1.06)
Postop. complications n(%)	25(2.42)
Transfusion n(%)	2(0.19)
ICU n(%)	19(1.84)
Adverse Event Notification n(%)	40(3.86)

All notifications of adverse events were classified as “Near Miss” and were related to documentation and prescription.

Complications (Table 3) were wound infection in 21 cases (2.03%), bleeding in seven (0.68%), bowel loop perforation in four (0.39%), bile duct injury in two (0.19%), biliary fistula in one (0.10%), and biliary stenosis in one case (0.10%).

**Table 3:** Complications.

Wound infection	21	2.0%
Bleeding	7	0.7%
Bowel loop perforation	4	0.4%
Bile duct injury (intraop. diagnosis)	2	0.2%
Biliary fistula	1	0.1%
Biliary tract stenosis	1	0.1%

**Figure 1:**
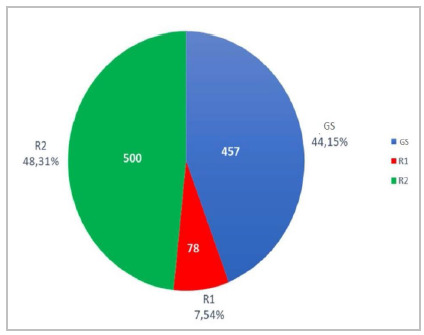
Distribution of patients according to group.

The three-group division resulted in 78 (7.5%) patients operated on with the participation of first-year residents (R1), 500 (48.3%) with the participation of second-year residents (R2), and 457 (44.2%) with the participation of only senior surgeons (Figure 1).

There were no statistically significant differences in the characteristics of patients operated between the three groups (Table 4).

**Table 4:** Patients’ characteristics by group.

	Surgeons		R2		R1		p
Age mean ± SD	51.4	± 14.9	52.6	± 14. 8	51.5	± 14.6	0.438
BMI mean ± SD	31.3	± 6.8	31.2	± 6. 8	31. 3	± 6.8	0.993
Sex n(%)							
Female	371	81. 0%	414	82.8%	65	83.3%	
Male	86	18.8%	86	17.2%	13	16.7%	0.775
Race							
White	208	45.4%	233	46.6%	40	5 1.3%	
Brown	191	41.7%	205	41. 0%	33	42.3%	
Black	52	11.4%	56	11.2%	5	6.4%	
Asian	6	1.3%	4	0.8%	0	0	
Native-American	0	0.0%	2	0.4%	0	0	0.491
SOI (Severity of Illness)							
1	449	98. 0%	488	97.6%	76	97.4%	
2	8	1.7%	12	2.4%	2	2. 6%	0.752
Previous laparotomy	7	1.5%	5	1. 0%	0	0	0.296
Preop. MRC	74	16.2%	57	11.4%	9	11.5%	0.085

**Figure 2:**
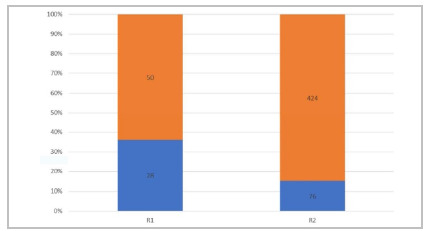
Graphic of the need for the assistants’ intervention.

Regarding the need for intervention by the assistant (senior surgeon), in the operations performed with the participation of residents, it was observed that it was more frequent, proportionally, in the R1 procedures, a fact that occurred in 28 occasions (35.9%), while 76 interventions (15.2%) in the group operated by R2 are needed (Figure 2, p <0.001).

Despite not showing statistical significance (p=0.053), the trend of longer hospital stays among patients operated on by the R1 group was clear, with an mean of 1.4 days versus 1.2 and 1.2 days for the R2 and Surgeons groups, respectively. There were no significant differences between the groups regarding the need of ICU and of blood transfusion (Table 5).

**Table 5:** Length of hospital stay - outcome.

	Surgeons		R2		R1		
	Mean	SD	Mean	SD	Mean	SD	p
Hospitalization days	1.2	0.8	1.2	1.4	1. 4	1.2	0.053
Surgery time (minutes)	50	25	53	23	60	21	<0.001
Total cost (US$)	1629.01	549.97	1616.62	712.73	1802.08	667.01	0.003
ICU	9	2. 0%	9	1.8%	1	1.3%	0.907
Transfusion	1	0.2%	1	0.2%	0	0	0.853

**Figure 3:**
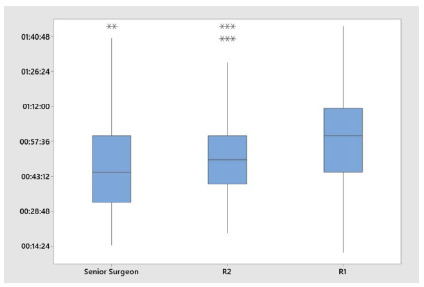
Operation time according to group.

However, there was a difference in the time of surgery (Figure 3), the mean duration of procedures performed by experienced surgeons being 50 minutes, and 53 and 60 minutes when there was participation of R2 and R1, respectively (p <0.001).

**Figure 4:**
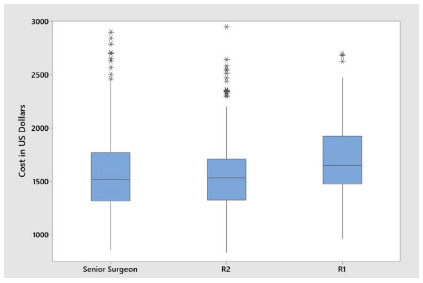
Total costs of hospitalization according to group.

There was also a difference as to hospitalization costs (p = 0.003), with a higher mean hospitalization cost for patients operated on by an R1, US$ 1,802.08, versus US$ 1,629.01 and US$ 1,616.62 in the procedures conducted by senior surgeons and R2, respectively (Figure 4).

There was no difference in conversion rates, complications, and reports of adverse events between the groups (Tables 6 and 7).

**Table 6:** Conversion rates and complications.

	Surgeons		R2		R1		p
Conversions	4	0.9%	4	0.8%	0	0	0.529
Complications Clavien I and II	16	3.5%	16	3.2%	1	1.3%	0.516
Complications Clavien III, IV and V	1	0.2%	2	0.4%	0	0	0.695
Intraop. complications	7	1.5%	6	1.2%	0	0	0.607
Postop. complications	10	2.2%	12	2.4%	1	1.3%	0.607
Adverse Event Notification	18	3.9%	18	3.6%	4	5.1%	0.815

**Table 7:** Complications’ distribution.

	Surgeons		R2		R1	
Wound infection	9	2, 0%	11	2.2%	1	1.3%
Bleeding	4	0.9%	3	0.6%	0	0
Bowel loop perforation	2	0.4%	2	0.4%	0	0
Bile duct injury (intraop. diagnosis)	1	0.2%	1	0.2%	0	0
Biliary fistula	1	0.2%	0	0	0	0
Biliary tract stenosis	0	0	0	0	1	1.3%
p = 0.928						

## DISCUSSION

The profile of operated patients followed the prevalence of chronic gallbladder calculous disease, more frequent in obese women in the sixth decade of life^17^. As for associated diseases, we opted for the use of SOI in the stratification of cases, since this score is commonly used to adjust the complexity of patients when it is intended to compare resource uses, rate of complications, and length of hospital stay between groups. Patients in our series can be considered of low severity, since the majority, 97.3%, had SOI level “1”.

The mean length of stay was 1.3 days, within what is usually reported in the literature^18^ ^-^ ^20^. The mean duration of our procedures was about 52 minutes, other works reporting values between 37 and 93 minutes in university hospitals^21^ ^-^ ^28^.

Our rate of conversion to open surgery (0.77%) was lower than the expected for this type of procedure, since the literature reports rates between 1% and 27.7%^29^ ^,^ ^30^. Similarly, our morbidity rates were also lower^31^ ^-^ ^34^. The main complications were related to the surgical wound, as expected.

We observed intraoperative bleeding in only 0.68% of the cases and more serious complications occurred only in isolated cases: bowel loop perforation in 0.39%, bile duct injury in 0.19%, and biliary fistula or stenosis in 0.1%.

Regarding hospitalization costs, the mean amount was R$ 6,299.10, which corresponds to US$ 1,636.10 when converted by the mean R$/US$ exchange rate in the period studied. It is common knowledge that absolute costs involve several locoregional and time factors, but for the sake of comparison, Traverso and Hargrave^35^ report a mean cost of US$ 2,490.00 for cholecystectomy in Seattle in the 1990s, while Lombardo et al.^36^ recorded a mean cost of US$ 227.00 in Mongolia, in 2017. 

When we carried out the stratification by groups, the greatest concern regarded the homogeneity between samples, since this is not a randomized study and, in theory, cases with factors that would add greater difficulty could not be performed by residents, interfering in the assessment of outcomes.

According to the literature, the variables that can be considered risk factors for conversions and perioperative complications are gender, BMI, previous abdominal surgery, comorbidities, and alteration of preoperative imaging and laboratory tests^29^ ^,^ ^30^ ^,^ ^32^. Therefore, we used these for the comparison between groups. However, there were no statistically significant differences between groups regarding these variables. Though lacking statistical difference, the presence of previous abdominal incisions prevented procedures from being performed by residents of the first year. With respect to patients undergoing preoperative MRC, there was also a slight tendency for them to be operated by double senior surgeons, rather than by R2 or R1 physicians (16.2% vs 11.4% and 11.5%).

Besides evaluating surgical outcomes, we compared groups regarding the rate of intervention of the senior surgeon in operations with the participation of residents. This was characterized by the reversal of roles between the assistant and the senior surgeon for more than 50% of the main procedure time. As anticipated, these exchanges occurred more often in procedures conducted by the first-year residents than by second-year ones (35.9% vs 15.2%).

We observed that the duration of surgery increased from the most to the least experienced surgeons (49min; 53min; 60min). We found an increase of about 20% of the surgical time when the procedure was performed with the participation of an R1; on the other hand, there was no significant difference between the senior team and the R2 group. Most authors make no distinction between the year of post-graduation the resident is. Papandria et al. refer an increase of 36% in the surgical time of procedures performed with the presence of residents, regardless of the year of training^26^. Johnson et al. show no difference in surgical time between the junior and senior residents but observed increased time compared with the group of assisting surgeons^24^. Our results may have been better, because although residents could perform all steps of the operation, in some cases in which there was no progression due to technical difficulty or anatomical condition, there was intervention by the tutor surgeon, to assist them or to finish the procedure. This interferes directly in the results displayed by these groups.

Regarding the length of hospital stay, although there was no statistical difference between groups, we observed a tendency for the group of patients operated on by R1s to stay longer in the hospital (1.4 vs 1.2 and 1.2). This may be explained by the longer surgical time and possible consequences, such as greater postoperative pain or the presence of nausea. Bisgard et al. refer that pain is responsible for an extra night of hospitalization in 26% to 41% of patients. One of the predictors of postoperative pain is the procedure time, trauma in the intraoperative manipulation, size of the incisions, blood loss or bile leakage, and calculi in the cavity^37^. Some authors also suggest that the presence of residents in the service may reduce the patients’ stay, perhaps due to the greater manpower available to perform bureaucratic tasks. However, they also make no distinction between the profile of operated patients and residents’ training degree^4^ ^,^ ^19^.

As for complication rates (regardless of type or severity), notifications of adverse events, need for blood transfusion, and use of ICU, we observed no differences between the three groups. This suggests that it is safe to perform laparoscopic cholecystectomy with the participation of residents in the first years of surgical training.

However, the literature is very conflicting on this subject. While Fahrner et al. and Ibrahim et al. demonstrate that residents have results like those of their tutors in terms of perioperative complications^4^ ^,^ ^22^, Kauvar et al. show that patients operated by junior residents have greater morbidity, with a decrease in this difference as they gain experience^8^. All three studies were not adjusted for confounding factors and need to be treated with caution.

Finally, we conducted an analysis of the total hospitalization costs. Traverso et al. reported that 60% of the total treatment costs occur in the operating room, with expenses related mainly to materials, disposable or not, drugs, and surgical team^35^. However, before discussing our results, we should note that the way we carried out the computation of the total cost can lead to biases, since it does not consider the exact time in the operating room, only the size of the procedure, in the apportionment of costs related to the operating room.

Another point concerns the human resources used in patient care, since it is difficult to measure or even separate the work performed by the attending physician from that performed by the resident one, and thus, reflect the difference in salary between the two categories in the cost. Residents perform a wide variety of tasks, such as filling out documents and reports, in addition to directly contributing to perioperative care. This results in time savings for the senior professional, who, in turn, usually receives nothing more for being involved in the training process of a new professional^24^. 

Considering the aspects discussed above, we observed that there was a cost difference between the group operated by first-year residents in relation to the second-year residents and the group operated only by senior surgeons. This difference was 11.47% more for the R1 group. There was no significant difference between the R2 and senior groups.

As the cost of operating room minute does not enter into this account, everything leads us to believe that the cost difference is probably due to the longer hospital stay, which, as previously discussed, may reflect the greater presence of postoperative symptoms / signs due to a longer procedure. Another possible hypothesis would be the greater concern about early discharging patients operated on by R1s.

Quantifying the financial cost of surgical resident education is no easy task. But it is evident that there must be a cost of training, especially in the first years. It is natural that, as the resident gains experience, this economic impact is mitigated in the hospital budget. On the other hand, the financial return for the community or even for the educational institution to train a highly competent surgeon is immeasurable.

We recognize some limitations of our work. This is a single-center study. The retrospective observational design, without randomization, is inherently associated with the risk of bias in the selection of cases, with less complex cases being prioritized for residents, especially in the first year. Future multicenter randomized controlled trials are essential to increase the strength of evidence of our findings.

## CONCLUSIONS

It is safe to perform laparoscopic cholecystectomy with the participation of residents, even in their first years of training, with no differences between groups as for complication rates (regardless of type or severity), adverse event reports, need of blood products transfusion, and use of ICU.

There is an additional cost of about 10% in the treatment of a patients operated with the participation of a first-year resident, there being no significant difference in the cost of the group operated by residents of the second year.

Teaching any trade requires time and dedication and usually involves some cost. In the health area, in addition to the factors mentioned, one cannot forget the concern with the safety of the main element of this process, the patient. But in this work, we were able to contribute with the idea that it is possible to prepare a new generation of surgeons who will be able to exercise their profession in a safe and effective manner, in a sustainable manner, even with the pressure of maintaining the efficiency of the institutions.
